# Sequence Similarity of *Clostridium difficile* Strains by Analysis of Conserved Genes and Genome Content Is Reflected by Their Ribotype Affiliation

**DOI:** 10.1371/journal.pone.0086535

**Published:** 2014-01-23

**Authors:** Hedwig Kurka, Armin Ehrenreich, Wolfgang Ludwig, Marc Monot, Maja Rupnik, Frederic Barbut, Alexander Indra, Bruno Dupuy, Wolfgang Liebl

**Affiliations:** 1 Technische Universität München, Department of Microbiology, Freising, Germany; 2 Laboratoire Pathogenèse des Bacteries Anaerobies**,** Institute Pasteur, Paris, France; 3 Institute of Public Health Maribor and University of Maribor, Faculty of Medicine and Centre of excellence Cipkebip, Ljubljana, Slovenia; 4 National Reference Laboratory for *Clostridium difficile*, Faculté de Médecine Pierre et Marie Curie and Hôpital Saint-Antoine, Assistance Publique-Hôpitaux de Paris, Paris, France; 5 Institute for Medical Microbiology and Hygiene, AGES – Austrian Agency for Health & Food Safety, Vienna, Austria; Charité, Campus Benjamin Franklin, Germany

## Abstract

PCR-ribotyping is a broadly used method for the classification of isolates of *Clostridium difficile*, an emerging intestinal pathogen, causing infections with increased disease severity and incidence in several European and North American countries. We have now carried out clustering analysis with selected genes of numerous *C. difficile* strains as well as gene content comparisons of their genomes in order to broaden our view of the relatedness of strains assigned to different ribotypes. We analyzed the genomic content of 48 *C. difficile* strains representing 21 different ribotypes. The calculation of distance matrix-based dendrograms using the neighbor joining method for 14 conserved genes (standard phylogenetic marker genes) from the genomes of the *C. difficile* strains demonstrated that the genes from strains with the same ribotype generally clustered together. Further, certain ribotypes always clustered together and formed ribotype groups, i.e. ribotypes 078, 033 and 126, as well as ribotypes 002 and 017, indicating their relatedness. Comparisons of the gene contents of the genomes of ribotypes that clustered according to the conserved gene analysis revealed that the number of common genes of the ribotypes belonging to each of these three ribotype groups were very similar for the 078/033/126 group (at most 69 specific genes between the different strains with the same ribotype) but less similar for the 002/017 group (86 genes difference). It appears that the ribotype is indicative not only of a specific pattern of the amplified 16S–23S rRNA intergenic spacer but also reflects specific differences in the nucleotide sequences of the conserved genes studied here. It can be anticipated that the sequence deviations of more genes of *C. difficile* strains are correlated with their PCR-ribotype. In conclusion, the results of this study corroborate and extend the concept of clonal *C. difficile* lineages, which correlate with ribotypes affiliation.

## Introduction

*Clostridium difficile* is a Gram-positive, anaerobic, spore forming bacterium. It is responsible for a broad spectrum of intestinal diseases ranging from self-limiting diarrhoea to life-threatening pseudomembranous colitis [1]. Nosocomial transmission and the use of antibiotics are the main drivers of *C. difficile* infection [2].

In the past 10 years *C. difficile* infections with increased disease severity and incidence emerged especially in Canada, U.S.A. and Western Europe [3], [4]. These outbreaks were traced back to a *C. difficile* strain typed as PCR-ribotype 027. Strains with PCR-ribotype 027 are mainly so-called hypervirulent strains. The initial association with a hypertoxigenicity phenotype is still controversial and not confirmed in all studies [5].

PCR ribotyping is a typing method that is currently becoming a standard for *C. difficile*. It is defined by differences in the 16S–23S rRNA intergenic spacer sequences present on multiple copies within a single *C. difficile* chromosome [6], [7]. The mechanisms behind the variations in the 16S–23S rRNA intergenic spacer sequences have been proposed to be slipped-strand mispairing and intra- and possibly interchromosomal homologous recombination [8].

The prevalence of PCR-ribotypes is geographically correlated [9]. Recent comparative genomic studies about *C. difficile* focus on strains with PCR-ribotype 027, 078 and strains related to these ribotypes [10]–[14]. About 50% of all *C. difficile* infections in North America and the United Kingdom belonged to PCR-ribotype 027 during 2003 to 2005, whereas on the other hand only 5% of all CDIs in 34 European hospitals were typed as PCR-ribotype 027 in 2008 [11]. The most common PCR-ribotypes in Europe are 014/020 (16%), 001 (9%) and 078 (8%) [15]. The emergence of specific *C. difficile* strains is not understood until now. Comparative phylogenetic studies with 75 isolates of *C. difficile* led to the identification of three apparently hypervirulent clonal lineages (PCR-ribotype 017, PCR-ribotype 027 and PCR-ribotype 078) as well as a fourth heterogeneous grouping [16], which is in line with other studies [12], [17]. However, the term hypervirulence must be used rather carefully [18].

The finding of clonal lineages indicates that *C. difficile* strains with the same PCR-ribotype appear to be somehow similar. However except for PCR ribotype 027, there is no study reporting in depth the similarity of the gene contents and gene sequences between strains with the same PCR-ribotype [11]. In this study we demonstrate correlations between the degree of sequence divergence of conserved genes of *C. difficile* strains and the strains’ PCR-ribotypes. Further, ribotype-specific genes for genomes with the same PCR-ribotype could be identified from whole-genome comparisons of 48 genome sequences of *C. difficile* strains, 27 of which were sequenced for this study, representing 21 different PCR-ribotypes.

## Methods

### DNA Sequencing and Genome Assembly

DNA was extracted of overnight culture growth in TY media, inoculated from one colony as described in [19]. Single-end multiplex libraries were created and the sequencing was performed using the Illumina HiSeq™ platform. The read length was 36-bp and 110-bp and all isolates were sequenced at least to an average coverage of 100-fold across the isolates. Sequencing reads were first scanned to remove the adaptator sequences and then were assembled into contigs using Velvet [20]. Thereafter, contigs were re-organized using Blast [21] alignment against the genome sequence of the reference *C. difficile* 630 strain. All contigs that did not match to the sequence of the reference strain were localized at the end of the contigs assembled to obtain a whole genome scaffold for each of the isolates. The sequenced *C. difficile* strains are deposited in the European Nucleotide Archive, the accession numbers are presented in the 2nd column of Table 1.

**Table 1:** Listing of accesion number, host and location of isolation of the 27 *C. difficile* strains sequenced for this study.

Strain	Accession number	Host	Location
E1	CAMD00000000 (WGS_scaffold) CAMD01000001-CAMD01000212 (contigs)	Human	Austria
E10	CAME00000000 (WGS_scaffold) CAME01000001-CAME01000293 (contigs)	Horse	Slovenia
E12	CAMZ00000000 (WGS_scaffold) CAMZ01000001-CAMZ01000353 (contigs)	Human	UK
E13	CAMF00000000 (WGS_scaffold) CAMF01000001-CAMF01000262 (contigs)	Human	Ireland
E14	CAMS00000000 (WGS_scaffold) CAMS01000001-CAMS01000319 (contigs)	Human	Hungary
E15	CAMM00000000 (WGS_scaffold) CAMM01000001-CAMM01000444 (contigs)	Human	France
E16	CAMH00000000 (WGS_scaffold) CAMH01000001-CAMH01000111 (contigs)	Human	France
E19	CAMO00000000 (WGS_scaffold) CAMO01000001-CAMO01000325 (contigs)	Human	UK
E23	CAMY00000000 (WGS_scaffold) CAMY01000001-CAMY01000264 (contigs)	Human	France
E24	CAMP00000000 (WGS_scaffold) CAMP01000001-CAMP01000063 (contigs)	Human	France
E25	CAMJ00000000 (WGS_scaffold) CAMJ01000001-CAMJ01000441 (contigs)	Human	France
E28	CAMX00000000 (WGS_scaffold) CAMX01000001-CAMX01000274 (contigs)	Human	France
E7	CAMV00000000 (WGS_scaffold) CAMV01000001-CAMV01000409 (contigs)	Human	Austria
E9	CAMU00000000 (WGS_scaffold) CAMU01000001-CAMU01000373 (contigs)	Horse	Canada
T10	CANB00000000 (WGS_scaffold) CANB01000001-CANB01000460 (contigs)	Human	Germany
T11	CAML00000000 (WGS_scaffold) CAML01000001-CAML01000409 (contigs)	Human	France
T14	CANC00000000 (WGS_scaffold) CANC01000001-CANC01000491(contigs)	Human	Ireland
T15	CAMK00000000 (WGS_scaffold) CAMK01000001-CAMK01000631 (contigs)	Human	Belgium
T17	CAMT00000000 (WGS_scaffold) CAMT01000001-CAMT01000449 (contigs)	Human	Hungary
T19	CANA00000000 (WGS_scaffold) CANA01000001-CANA01000247 (contigs)	Human	France
T20	CAMC00000000 (WGS_scaffold) CAMC01000001-CAMC01000210 (contigs)	Human	Ireland
T22	CAMI00000000 (WGS_scaffold) CAMI01000001-CAMI01000332 (contigs)	Human	Hungary
T23	CAMN00000000 (WGS_scaffold) CAMN01000001-CAMN01000293 (contigs)	Human	Italy
T42	CAMQ00000000 (WGS_scaffold) CAMQ01000001-CAMQ01000096 (contigs)	Human	Italy
T3	CAMW00000000 (WGS_scaffold) CAMW01000001-CAMW01000275 (contigs)	Human	Italy
T5	CAMB00000000 (WGS_scaffold) CAMB01000001-CAMB01000227 (contigs)	Human	Italy
T6	CAMR00000000 (WGS_scaffold) CAMR01000001-CAMR01000373 (contigs)	Human	Hungary

### Reciprocal Best Hits BLAST

A variation of the BLAST [21] reciprocal-best-hit method was used to identify orthologous CDSs (coding sequences) between two genomes. With the standard BLAST reciprocal-best-hit method two CDSs, C1 and C2 (from genome G1 and G2 respectively) are considered to be orthologous if and only if C2 is the best BLAST hit when G1 is used as the query sequence and all CDSs in G2 are used as the database, and (b) C1 is the best BLAST hit when G2 is used as query sequence and all CDS of G1 are used as the database. We modified the method by extending best hit to best hits while establishing threshold 1 and threshold 2. Threshold 1 says one hit can be grouped as one of the best hits if and only if the bit-score of the hit is equal or smaller than e.g. 80% of the maximal bit-score. Threshold 2 is analogous to threshold 1, except that it refers to the length of the query CDS and is defined as the percentage of the query sequence. Threshold 1 and 2 were set to 0.8 BLAST was used with the default parameters and an E-value cut-off of 10^−5^ and the filter-option was set to false. The BLAST release 2.2.22 was locally installed. The BLAST reciprocal best hits method was implemented in Java. The results of the reciprocal best hits BLAST were stored in a MySQL database. That method is named reciprocal best hits BLAST.

In our dataset the genome sequences of six *C. difficile* genomes were not annotated (see Table 2). To use these strains we assigned functions to ORFs (open reading frames) by using the reciprocal best hits BLAST. First, ORFs (open reading frames) were predicted in all six genomes with the software GLIMMER [22]–[24]. In total, three reference genomes (*C. difficile* R20291, *C. difficile* CD196 and *C. difficile* 630) were used for each annotated genome (Table 2). Orthologous sequences were computed with reciprocal best hits BLAST for all predicted ORFs of each organism and all CDSs of each reference genome like described above. The reciprocal best hits BLAST was computed using translated nucleotide BLAST (tblastx) with default parameters, expect an E-value cut-off of 10^−5^ and the filter-option was set to false, threshold 1 and 2 were set to 0.8.

**Table 2:** Ribotype, toxinotype and accession number of 21 *C. difficile* strains with previously reported genome sequences used in this study.

Strain	Accession number	PCR-ribotype	Toxinotype
BI1*	FN668941.1 (chromosome) FN668943.1 (chromosome) FN668942.1 (plasmid)	027	III
BI9*	FN668944 (chromosome)	001	0
2007855*	FN665654.1 (chromosome)	027	III
630	AM180355.1 (chromosome) AM180356.1 (plasmid)	012	0
CD196	FN538970.1	027	III
CF5*	FN665652.1	017	VIII
M120*	FN665653.1	078	V
M68*	FN668375.1	017	VIII
R20291	FN545816.1	027	III
ATCC 43255	CM000604.1	087	0
CIP-107932	CM000659 (WGS_scaffold) ABKK02000001-ABKK02000055 (contigs)	027	III
QCD-23m63	CM000660 (WGS_scaffold) ABKL02000001-ABKL02000061 (contigs)	078	V
QCD-32g58	CM000604 (WGS_scaffold) NZ_AAML04000001-NZ_AAML04000016 (contigs)	027	III
QCD-37x79	CM000658 (WGS_scaffold) NZ_ABHG02000001.1 - NZ_ABHG02000031.1 (contigs)	027	III
QCD-63q42	CM000637 (WGS_scaffold) ABHD02000001-ABHD02000060 (contigs)	001	0
QCD-66c26	CM000441 (WGS_scaffold) ABFD02000001-ABFD02000031 (contigs)	027	III
QCD-76w55	CM000661 (WGS-scaffold) ABHE02000001-ABHE02000066 (contigs)	027	III
QCD-97b34	CM000657 (WGS_scaffold) ABHF02000001-ABHF02000060 (contigs)	027	III
NAP07	GG770744-GG770776 (WGS_scaffold) ADVM01000001-ADVM01000100 (contigs)	078	V
NAP08	GG770710-GG770733 (WGS_scaffold) ADNX01000001-ADNX01000111 (contigs)	078	V
CD002	CAMG00000000 (WGS_scaffold) CAMG01000001-CAMG01000071 (contigs)	002	0

Strains marked with an asterisk are not annotated. The ribotypes of these strains were calculated using their GenBank data.

To determine the core genomes of any set of *C. difficile* strains, orthologous CDSs were identified between all CDSs in the set, like described before. If one CDS had an orthologous CDS in every strain of the set the CDS was assigned to the core genome. Different core genomes or the whole genomes, in case no core genome could be computed, were compared to detect CDSs that are specific for a special set.

The reciprocal best hits BLAST was computed using nucleotide BLAST (blastn) with default parameters, expect E-value cut-off of 10^−5^ and the filter-option was set to false, threshold 1 and 2 were set to 0.8.

### Distance Matrices

Fourteen gene sequences encoding highly conserved proteins () were used for distance matrices computations. The respective protein primary structures are commonly used besides the rRNA sequences as markers in comprehensive phylogenetic studies of the organisms [25]. The nucleotide sequences of the 14 gene sequences from strain CD630 were download from the UniProt database [26]. To detect the corresponding gene sequences in the remaining *C. difficile* strains, nucleotide sequences comparisons with BLAST were performed using as query the nucleotide sequences of the 14 CD630 genes and as database all whole genome sequence of all analysed *C. difficile* strains in the study. For each of the 14 genes, the group of detected nucleotide sequences was aligned using the ARB program [27] using processed seed databases as templates. Distance matrices-based dendrograms were obtained applying the neighbor joining method implemented in ARB. The distance matrices contain always the normalized hamming distances. The normalized hamming distance between two sequences is the number of different characters divided by the number of comparisons. To estimate the root of the dendrograms, the corresponding conserved genes from close relatives of *C. difficile* were included.

## Results

### Distance Matrices of Conserved Genes Correlate with Ribotypes

In the current study we primarily investigated correlations between 14 conserved genes and 21 different ribotypes of 48 *C. difficile* strains with completely sequenced genomes, see Table 2 and Table 3. These 14 conserved genes were investigated to see if the ribotype is reflected in their sequences. The lengths of the 14 different conserved genes for *ATPase alpha, ATPase alpha V-type, ATPase beta, ATPase beta V-type, RNA polymerase A, RNA polymerase B, RNA polymerase C, DNA gyrase A, DNA gyrase B, elongation factor G, heat shock protein 60, heat shock protein 70, initiation factor, recombinase*, range from 948 to 3717 nucleotides (Table 4).

**Table 3:** Phenotypic description of the 27 *C. difficile* strains sequenced for this study.

Strain	PCR-ribotype (agarose)	PCR-ribotype (WEBRIBO)	Toxinotype	Fluoroquinolone resistance
E1	126	126	V	S
E10	033	033	XIa	S
E12	106	106	0	R
E13	017	017	VIII	R
E14	014	014/0	0	S
E15	075	075	III	S
E16	001*	577	0	S
E19	036	578	X	S
E23	001/072	241	0	S
E24	020	020	0	S
E25	005	005	0	S
E28	012	012	0	S
E7	053	053	0	R
E9	009	009	NA	S
T10	001/072	001	0	R
T11	075	075	III	S
T14	106	106	0	R
T15	005	005	0	I
T17	025	665	0	R
T19	057	237	0	I
T20	078	078	V	S
T22	No data	No data	0	ND
T23	019	019	IX	S
T42	No data	No data	No data	ND
T3	012	012	0	I
T5	126	126	V	R
T6	014	014/0	0	I

The strain names, PCR-ribotypes, toxinotypes, and fluoroquinolone resistance of 27 *C. difficile* strains used and sequenced for this study are listed. The PCR-ribotype was determined agarose-based [6] and WEBRIBO-based [35]. PCR-ribotype 001* is similar but not identical to PCR ribotype 001/072. The toxinotype of strain E9 could not be determined because it has no ToxinA and ToxinB genes, but genes for the binary toxins.

**Table 4:** Characteristics of the 14 conserved genes used for the computation of the distance matrices.

Conserved gene	Locus tag	Gene ID	Length	Maximal Hamming distance
ATPase Alpha (atpA)	CD630_34700	4914804	1503	1.33
ATPase Alpha V-Type (atpA)	CD630_29560	4913755	1779	1.35
ATPase Beta (atpD)	CD630_34680	4914802	1395	1.15
ATPase Beta V-Type (atpB)	CD630_29550	4913754	1374	1.97
RNA polymerase A (rpoA)	CD630_00980	4913146	948	1.59
RNA polymerase B (rpoB)	CD630_00660	4914216	3717	2.21
RNA polymerase C (rpoC)	CD630_00670	4914217	3486	2.84
Gyrase A (gyrA)	CD630_00060	4915790	2427	2.02
Gyrase B (gyrB)	CD630_00050	4915789	1902	2.79
Initiation factor	CD630_13090	4914468	1941	1.2
Recombinase (recap)	CD630_13280	4914615	1047	2.29
Heatshock protein 70 (dnaK)	CD630_24610	4916451	1848	1.25
Heatshock protein 60 (groL)	CD630_01940	4915463	1629	2.71
Elongation factor G (fusA)	CD630_00700	4914220	2067	1.21

The locus tag, length values and gene IDs listed in the table were taken from the genome data of *C. difficile* strain CD630. The maximal hamming distance is a measure for the dissimilarity of the 14 conserved genes in all analysed 48 *C. difficile* strains.

The diversity of the 48 *C. difficile* strains was studied by distance matrix-based clustering. To figure out the differences between the sequences, the simplest method to compute distances between sequences was used (Neighbor Joining). For each highly conserved gene a distance matrix was computed (see section on Distance matrices below). Table 4 shows that the maximal hamming distances ranged from 1.15 (*ATPase beta* gene) to 2.84 (*RNA polymerase C* gene). For the gene *ATPase beta* not more than 15 of overall 1395 nucleotides differ and for the gene *RNA polymerase C* not more than 78 of overall 3486 nucleotides. Thus, the nucleotide sequence divergence for each of the 14 investigated conserved genes is generally low, differing only in few nucleotides among the 48 studied *C. difficile* genomes. Considering the high degree of similarity of the nucleotide sequences, the corresponding protein sequences were neglected.

**Figure 1:**
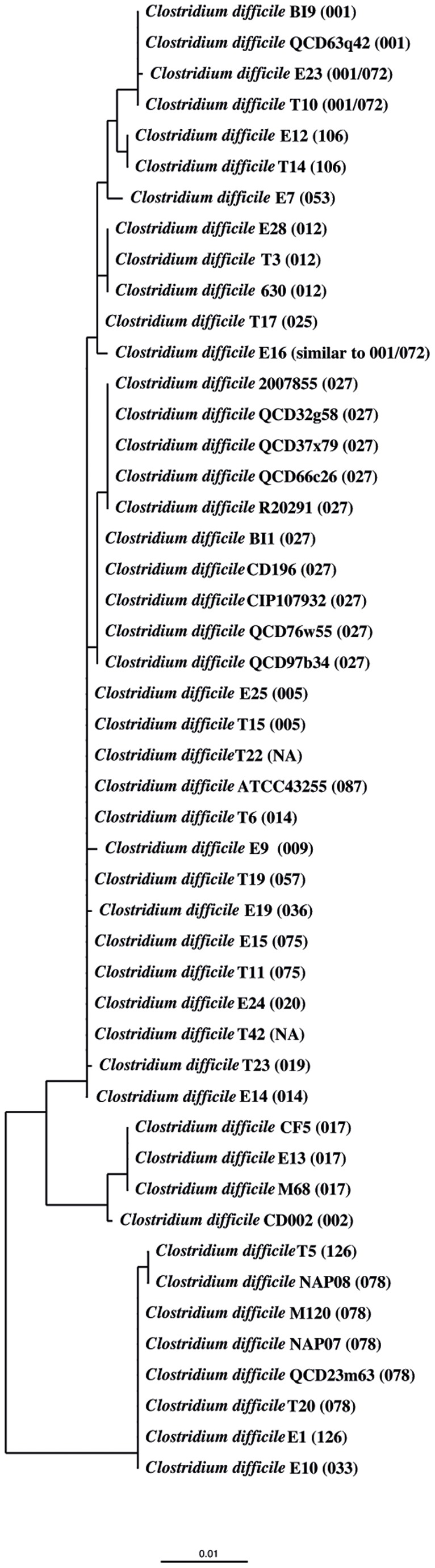
DNA gyrase A gene-based dendrogram. Neighbor joining dendrogram reflecting the similarity for 48 *C. difficile* strains based on the gene for DNA gyrase A. The distance matrix was computed using the Hamming distance with the DNA gyrase A genes from the 48 *C. difficile* isolates containing this gene. The strains always cluster together according to their PCR-ribotype. The PCR-ribotype is indicated in brackets. The strains with ribotype 027 sub-cluster into two different groups.

To visualize the results, dendrograms were computed for each distance matrix. Each of the 14 computed dendrograms showed that *C. difficile* strains with the same PCR-ribotype cluster together (Fig. 1, and Fig. S1, S2, S3, S4, S5, S6, S7, S8, S9, S10, S11, S12, S13). In addition, the dendrograms of all conserved genes showed further clustering of PCR-ribotype groups. Strains with PCR-ribotype 078, 033 and 126 always clustered together, as well as strains with PCR-ribotypes 017 and 002. Strains from ribotype 027 usually represent the independent lineage or cluster together with ribotype 019, 036 and 075.

The dendrogram reflecting gyrase A (Fig. 1) shows a sub-clustering of the ten strains with PCR-ribotype 027. Five strains, 2007855, QCD37x79, QCD66c26, R20291 and QCD32g58, are grouped into one cluster whereas the other five ribotype 027 strains, QCD76w55, BI1, ATCC43255, CD196 and CIP107932, formed another cluster. These two sub-clusters only differ by one nucleotide at position 245, the first group showing thymine at that position and the second group cytosine. The amino-acid sequence also differs at the corresponding position on the protein sequence level group 1 having isoleucine and group 2 threonine at that position. It is noteworthy that these clusters correlate with the strains’ fluoroquinolone resistance [11].

The two sub-clusters of the ten strains with PCR-ribotype 027 were also found in a dendrogram calculated from the concatenated sequences of all 14 conserved genes. Additionally, in that dendrogram strains with the same ribotypes also form clusters and the strains with the PCR-ribotypes 033, 078 and 126 formed one cluster, as well as the two PCR ribotypes 002 and 017 (Fig. S14).

### Gene Content Analysis with Reciprocal BLAST Reveals that Some Ribotypes are more Similar than others

According to the PCR-ribotype and conserved gene-based clustering of the *C. difficile* strains, we defined a core genome from all genes for each cluster using reciprocal best hits BLAST. A cluster is defined to contain *C. difficile* strains with the same PCR-ribotype. Eleven PCR-ribotypes are represented by exactly one genome and each of them builds exactly one cluster. These ribotypes are 002, 009, 019, 020, 025, 033, 036, 053, 057, 087, and 001* (similar but not identical to 001). Ten other PCR-ribotypes (001, 005, 012, 014, 017, 027, 075, 078, 106 and 126) are represented by 2 or more genomes. The cluster called 001 comprises strains with ribotype 001 and 001/072. Each core genome represents the set of all orthologous CDSs shared by all members of the cluster. Core genomes could only be computed of ribotypes, which were represented by two or more strains. For those ribotypes, which were represented by merely one strain, the whole genome instead of a core genome was included in the genome comparisons. Core genome and whole genome comparisons allowed the search for genes which are specific for (i.e. exclusively shared by) all members of a given cluster or cluster group.

The size of each core genome depends on the number and diversity of the considered genomes. The number of genes determined for the 10 different core genomes ranges from 2299 to 3617 genes. The most comprehensive cluster (10 of 48 strains) in our dataset combines the PCR-ribotype 027 strains. This group shares the smallest core genome comprising 2299 genes. The largest core genome with 3617 genes was found for the PCR ribotype 075 with the strains E15 and T11. The remaining groups have two (ribotype 005, 014, 106 and 126), three (ribotype 017, or 012), four (ribotype 001) or five (ribotype 078) representatives and core genome sizes ranging from 2415 to 3589 genes.

The percentage of genes shared between the core genomes or the whole genomes of each possible comparison between two groups of PCR-ribotypes varied from 75.9% (009/033) to 99.7% (078/126). More than 98% of all genes of the core genome or the whole genome of one PCR-ribotype were identified in the comparison of PCR-ribotype 012 with 053, 014 with 001*, 001 with 053, and 014 with 020. Furthermore more than 97% genes were found in 8 other genome comparisons. 2 other comparisons resulted in less than 80% shared genes. The lowest percentage of shared genes, 77.6% und 78%, were identified upon comparison of the whole genomes of PCR-ribotype 087 with 577 and the whole genome of 009 with the core genome 126, respectively.

The core genome of the group with PCR-ribotype 078 revealed 5 specific genes when compared with the core genome of ribotype 126 and 69 specific genes when compared with the whole genome of strain E10 (PCR-ribotype 033). The 5 specific genes for ribotype 078 in comparison to ribotype 126 are 2 hypothetical proteins (locus_tag = CdifQCD-2_020200008002, locus_tag = CdifQCD-2_020200004284), integrase (locus_tag = CdifQCD-2_020200004815), sigma-54 dependent regulatory protein (locus_tag = CdifQCD-2_020200006932) and sigma-54-dependent transcriptional activator (locus_tag = CdifQCD-2_020200016221).

These small numbers of specific genes support the clustering of these three ribotype strains found by the comparative analyses of the 14 conserved genes described above.

The second group that always clustered together in the analysis of conserved genes were ribotypes 017 and 002. In the course of comparison of their core genomes and whole genome, 86 specific genes were identified for PCR-ribotype 017.

### Distance Matrix Computation of Toxin Genes

In all 48 analysed *C. difficile* genome sequences 12 different genes were identified that have been associated with virulence and 21 genes associated with toxin activity. The genes associated with toxin (purely annotation-based selection, knowing that some of these genes are unlikely to be virulence-related) comprise these gene designations: toxin-antitoxin system, antitoxin component Xre family (BN189_1150005); putative toxin-antitoxin system, toxin component, Bro family (BN180_2630008); toxin secretion/phage lysis holin (CdifA_020200008837); toxin-antitoxin system, toxin component, RelE family (BN180_2600020); ADP-ribosyltransferase enzymatic component (*cdtA*) (CD196_2444); ADP-ribosyltransferase binding protein (*cdtB*) (CD196_2445), binary toxin regulatory gene, LytTR family (*cdtR*) (BN175_1830013); toxin A (*tcdA*) (CDR20291_0584); toxin B (*tcdB*) (CD196_0600); negative regulator of toxin gene expression (*tcdC*) (CDR20291_0585); putative cell wall hydrolase protein (*tcdE*) (CDR20291_0583), epsilon-antitoxin (BN182_1810006); zeta-toxin (BN182_1810005); neurotoxin Cex100 (BN168_390016); enterotoxin, EntD (BN175_600004); iota toxin component Ia (BN166_1780002); iota toxin component Ib (BN166_1780003); C2 toxin component I (CdifQCD-2_020200012999) and C2 toxin component II (CdifQCD-2_020200013004). The gene for binary toxin A is annotated differently in the genomes of our data set. It is annotated as binary toxin A, C2 toxin component I and iota toxin component Ia. In accordance to our specifications these CDSs are orthologous. The same holds true for the CDSs for binary toxin B, C2 toxin component II and iota toxin component Ib.

BLAST comparisons of all genes that are associated with toxin activity were performed against the National Center for Biotechnology Information (NCBI) non-redundant database to see if these genes are similar to genes where toxin activity was described. In addition to toxin A, toxin B and binary toxin no other gene for which association with toxin activity was reported could be detected.

**Figure 2:**
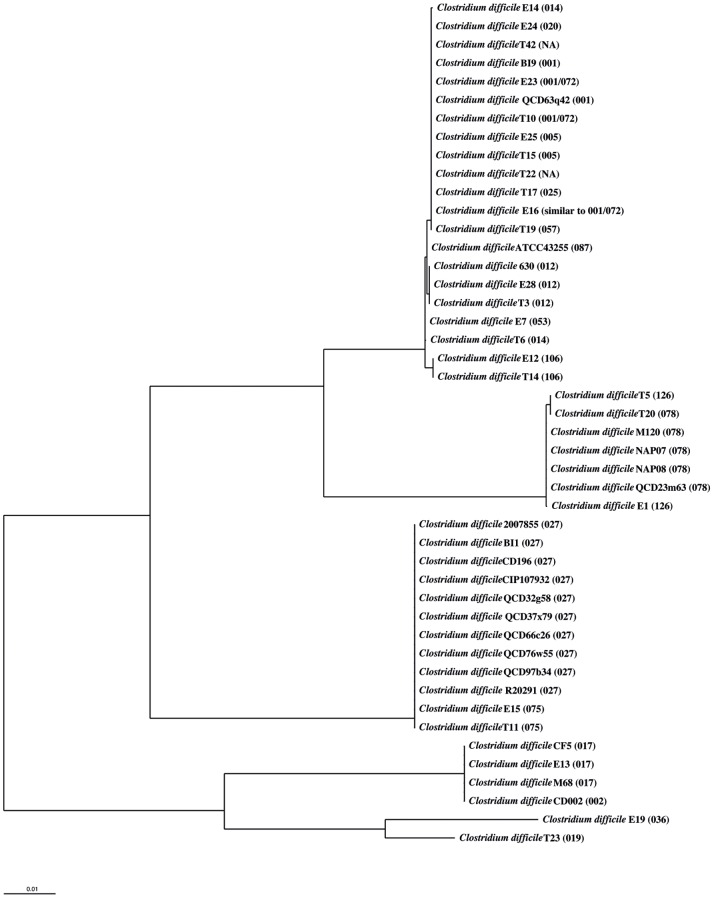
Toxin B gene-based dendrogram. Neighbor joining dendrogram reflecting the similarity for 46 *C. difficile* strains based on the gene for Toxin B. The distance matrix was computed using the Hamming distance with the Toxin B genes from the 46 *C. difficile* isolates containing this gene. The strains always cluster together according to their PCR-ribotype. The PCR-ribotype is indicated in brackets.

Differences in toxin genes of *C. difficile* strains as found by comparisons of the pathogenicity loci have been used for differentiation purposes (toxinotypes; [28]). Therefore, analogous to the distance matrices computations described above for 14 conserved genes, dendrograms were also computed for the genes for toxin B (*tcdB*), negative regulator of toxin gene expression (*tcdC*), binary toxin A (*cdtA*), binary toxin B (*cdtB*) and the binary regulatory protein (*cdtR*) (Fig. 2, and Fig. S15, S16, S17, S18). For the toxin A gene (*tcdA*) no dendrogram was computed because the sequences from 20 of the 48 strains due to difficulties in completing sequences containing repeats with Illumina technology had gaps in the toxin A sequence and the result would then be falsified. The dendrograms for these genes show that strains with the same ribotype cluster and support the same groups of PCR-ribotypes as described above for the conserved genes.

Furthermore we computed a distance matrix with the gene annotated as putative virulence associated protein E, *virE* (CD196_1450). This gene was included in the analysis based on this annotation, being well aware that its connection with virulence of *C. difficile* is uncertain and that it is not present in every analysed strain. The *virE*-orthologous gene is present in the genomes with PCR-ribotype 009, 017, 025, 027, 075 and 087 (19 strains). The putative virulence-associated protein E belongs to the family VirE, to the Clan P-loop NTPase (CL0023) and its gene is 1417 nucleotides long.

**Figure 3:**
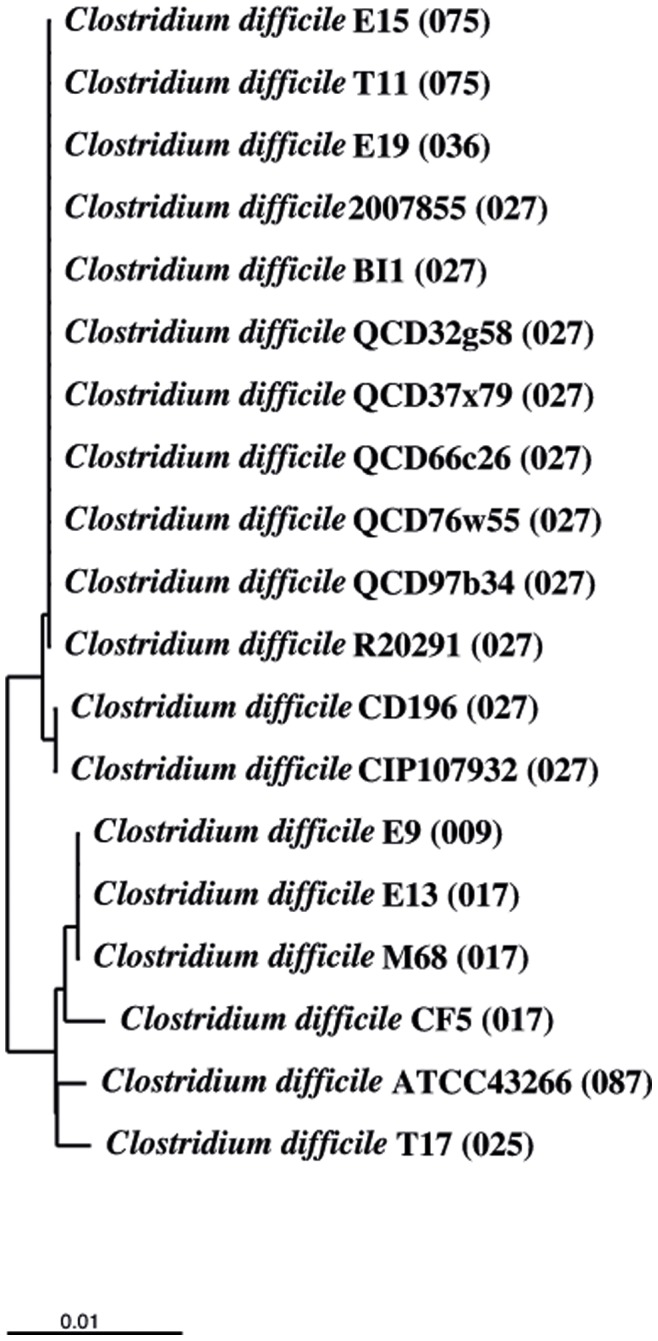
Virulence associated protein e gene-based dendrogram. Neighbor joining dendrogram reflecting the similarity for 19 *C. difficile* strains based on the gene for virulence associated protein E. The distance matrix was computed using the Hamming distance with the virulence associated protein E genes from the 19 *C. difficile* isolates containing this gene. The strains always cluster together according to their PCR-ribotype. The PCR-ribotype is indicated in brackets. The strains with ribotype 027 sub-cluster into two different groups.

As described above for the toxin-associated genes and the 14 conserved genes we also computed a dendrogram for the *virE* gene (Fig. 3). Similar to the conserved gyrase A gene we detected a sub-clustering of strains with PCR-ribotype 027 in two groups. Strains CD196 and CIP107932 represented a common group, while strains BI1, QCD-32g58, QCD-37x79, QCD-97b34, QCD-66c26, QCD-76w55, 2007855, R20292, T11, E19 and E15 clustered in the other group. The first group only differs by two nucleotides from the second group. The nucleotide at position 772 is cytosine in the first group but adenine in the second group, resulting in threonine or asparagine residues, respectively, at the corresponding amino acid sequence positions. The other difference is at nucleotide position 992, were either cytosine (group 1) or thymine (group 2) is found, with no consequence for the amino acid sequence.

## Discussion

PCR-ribotyping is a common method to group *C. difficile* strains and the standard method in laboratorys Europe-wide. Recently, a module-like structure of PCR-ribotype defining sequences was identified [8], and studies based on strains from a limited number of ribotypes have indicated that the phylogenetic diversity of *C. difficile* is reflected by the PCR-ribotypes [12], [16], [17], [29]. Another study based on *C. difficile* strains with different ribotypes reported that the phylogenetic diversity is best reflected by the MLST [30]. Our work complements and corroborates these studies by analysing in depth for the first time the correlations between the PCR-ribotype and the gene content and the relatedness of conserved genes among the fully sequenced genomes of a total of 48 *C. difficile* strains. Importantly, our study includes the genomes of strains belonging to a broad variety of different ribotypes. To this end, we present for the first time a gene content analysis of *C. difficile* strains representing 21 different ribotypes. In contrast to existing studies [10]–[14], [16], [17], [29] we do not focus on strains with ribotypes 027 or 078, and in contrast to a recent study on MLST comparison of various *C. difficile* strains from different ribotypes [30] we compared fully sequenced genomes.

Despite the development of new typing methods for *C. difficile* like whole-genome SNP typing it becames apparent that especially capillary ribotyping is cheaper and faster. Additionally the discrimatory power of whole-genome SNP and capillary ribotyping is identical [31], [32]. Our results demonstrate that the PCR ribotype is not only reflected by the sequences between the 16S and 23S rRNA but also by 21 different genes. Therefore, whole genome SNP approaches do not automatically represent the ultimate pathogen typing method [31].

By computing distance matrices with neighbor joining for 14 conserved genes we found that the nucleotide sequence differences between the representatives of each conserved gene are small but these small differences are always correlated with the PCR-ribotypes of the *C. difficile* strains. This observation emphasizes that the assignment of the PCR-ribotype is a very suitable method to group *C. difficile* strains.

The dendrogram computed with the gene DNA gyrase A showed that the 10 *C. difficile* strains with ribotype 027 analysed here differ at one nucleotide sequence position where either a thymine or a cytosine is found, resulting in either a threonine or isoleucine residue, respectively, at the corresponding GyrA amino acid sequence position. That *gyrA* mutation is associated with fluoroquinolone resistance and was identified to be one of the factors that correlate with two distinct epidemic lineages of *C. difficile* strains with ribotype 027 [11].

We identified two groups of *C. difficile* that always clustered together in the distance matrix analyses, one group comprising PCR-ribotypes 033, 078, and 126, and a second group comprising PCR-ribotypes 002 and 017. The clustering for strains with PCR-ribotypes 033, 078 and 126 has already been observed before [16], [30], [33]. PCR-ribotype 126 differs only by the loss of one single band on the amplified DNA band pattern of PCR-ribotype 078 [34]. The seven strains with PCR-ribotypes 078 and 126 have the same toxinotype V, while the strain with PCR-ribotype 033 has toxinotype XIa. It has been reported that these strains together with strains from the ribotypes 045, 066 and 193 belong to the same evolutionary lineage [30]. The four strains with PCR-ribotypes 002 and 017 have also different toxinotypes (0 and VIII). The computation of the genes specific for certain groups of PCR-ribotypes confirmed the clustering of all two groups. The PCR-ribotype groups 033/078 and 078/126 had only few specific genes meaning they have few differences in the gene content. In this context it is interesting to note that in practise 078 and 126 strains are not always easily differentiated and therefore are sometimes designated as ribotype 078/126.

The genome database annotation of the gene *virE* as “putative virulence-associated protein E” is suggestive of a possible role in virulence. Despite the fact that the function of *virE* has not been determined in *C. difficile* and hence it is not clear if it is really associated with virulence, we have included the *virE* sequences in the comparative analysis of this study, yielding an interesting result. *VirE* and DNA gyrase A were the only analysed CDS showing a sub-clustering of all PCR-ribotype 027 strains into two groups. In the *virE*-based dendrogram, group 1 contains strains CD196 and CIP107932, both isolated in France. The second group contains strains 2007855, BI1, QCD32g58, QCD37x79, QCD66c76, QCD76w55, QCD97b34, R20291 with PCR-ribotype 027, strains E15 and T11 with PCR-ribotype 075 and E19 with PCR-ribotype 001*. Strains with PCR-ribotype 027 from the second group were isolated, as far as known, in Canada, the United Kingdom and USA. The other three strains from the second group were isolated in France and the United Kingdom. There could be an association between PCR-ribotype 027 and the country of isolation that is reflected by the gene *virE*.

Regarding all specific genes and all genes of each core genome no gene could be identified that is associated with toxin activity except the familiar genes for toxin A, toxin B, toxin C and the binary toxins. Hence no gene associated with toxin activity could be correlated only with so called hypervirulent *C. difficile* strains.

It has been reported recently that PCR ribotype 027 is very similar to the ribotypes 016, 036 and 176 [30]. In our analysis the strain with ribotype 036 is always allocated to the same node except in the dendrograms for the genes *tcdB* and *gyrA*. Strains with the ribotypes 016 and 176 are not contained in our dataset.

In conclusion, this report demonstrates that the PCR-ribotype is correlated with differences in the sequences of conserved genes. Thus it appears that the PCR-ribotype is indicative not only of a specific pattern of the 16S-23S rRNA intergenic spacer sequences, but also reflects specific differences in the nucleotide sequences of numerous genes such as the genes studied here. Perhaps the sequence deviations of many more *C. difficile* genes are correlated with the PCR-ribotypes of the corresponding strains.

## Supporting Information

Figure S1 **ATPase α V-Type gene-based dendrogram.** Neighbor joining dendrogram reflecting the ATPase α V-Type-based species similarity for all 48 analyzed *C. difficile* strains. The distance matrix was computed using the Hamming distance with the ATPase α V-Type genes from the 48 *C. difficile* isolates. The PCR-ribotype is indicated in brackets. All *C. difficile* strains cluster together according to their PCR ribotype.
(EPS)

Figure S2 **ATPase α F1F10 gene-based dendrogram.** Neighbor joining dendrogram reflecting the DNA ATPase α F1F10-based species similarity for all 48 analyzed *C. difficile* strains. The distance matrix was computed using the Hamming distance with the ATPase α F1F10 genes from the 48 *C. difficile* isolates. The PCR-ribotype is indicated in brackets. All *C. difficile* strains cluster together according to their PCR ribotype.
(EPS)

Figure S3 **ATPase β V-Type gene-based dendrogram.** Neighbor joining dendrogram reflecting the ATPase β V-Type-based species similarity for all 48 analyzed *C. difficile* strains. The distance matrix was computed using the Hamming distance with the ATPase β V-Type genes from the 48 *C. difficile* isolates. The PCR-ribotype is indicated in brackets. All *C. difficile* strains cluster together according to their PCR ribotype.
(EPS)

Figure S4 **ATPase β F1F10 gene-based dendrogram.** Neighbor joining dendrogram reflecting the DNA ATPase β F1F10-based species similarity for all 48 analyzed *C. difficile* strains. The distance matrix was computed using the Hamming distance with the ATPase β F1F10 genes from the 48 *C. difficile* isolates. The PCR-ribotype is indicated in brackets. All *C. difficile* strains cluster together according to their PCR ribotype.
(EPS)

Figure S5 **Elongation factor G gene-based dendrogram.** Neighbor joining dendrogram reflecting the Elongation factor G gene-based species similarity for all 48 analyzed *C. difficile* strains. The distance matrix was computed using the Hamming distance with the Elongation factor G genes from the 48 *C. difficile* isolates. The PCR-ribotype is indicated in brackets. All *C. difficile* strains cluster together according to their PCR ribotype.
(EPS)

Figure S6 **DNA gyrase B gene-based dendrogram.** Neighbor joining dendrogram reflecting the DNA gyrase B gene-based species similarity for all 48 analyzed *C. difficile* strains. The distance matrix was computed using the Hamming distance with the DNA gyrase B genes from the 48 *C. difficile* isolates. The PCR-ribotype is indicated in brackets. All *C. difficile* strains cluster according to their PCR ribotype.
(EPS)

Figure S7 **Heatshock protein 60 gene-based dendrogram.** Neighbor joining dendrogram reflecting the heat shock protein 60 gene-based species similarity for all 48 analyzed *C. difficile* strains. The distance matrix was computed using the Hamming distance with the heat shock protein 60 genes from the 48 *C. difficile* isolates. The PCR-ribotype is indicated in brackets. All *C. difficile* strains cluster according to their PCR ribotype.
(EPS)

Figure S8 **Heatshock protein 70 gene-based dendrogram.** Neighbor joining dendrogram reflecting the heat shock protein 70 gene-based species similarity for all 48 analyzed *C. difficile* strains. The distance matrix was computed using the Hamming distance with the heat shock protein 70 genes from the 48 *C. difficile* isolates. The PCR-ribotype is indicated in brackets. All *C. difficile* strains cluster according to their PCR ribotype.
(EPS)

Figure S9 **Initiation factor gene-based dendrogram.** Neighbor joining dendrogram reflecting the Initiation factor gene-based species similarity for all 48 analyzed *C. difficile* strains. The distance matrix was computed using the Hamming distance with the Initiation factor genes from the 48 *C. difficile* isolates. The PCR-ribotype is indicated in brackets. All *C. difficile* strains cluster according to their PCR ribotype.
(EPS)

Figure S10 **Recombinase gene-based dendrogram.** Neighbor joining dendrogram reflecting the similarity for all 48 analyzed *C. difficile* strains based on the gene for recombinase. The distance matrix was computed using the Hamming distance with the recombinase genes from all 48 *C. difficile* isolates. The strains always cluster according to their PCR ribotype. The PCR-ribotype is indicated in brackets.
(EPS)

Figure S11 **RNA polymerase A gene-based dendrogram.** Neighbor joining dendrogram reflecting the similarity for all 48 analyzed *C. difficile* strains based on the gene for RNA polymerase A. The distance matrix was computed using the Hamming distance with the RNA polymerase A genes from all 48 *C. difficile* isolates. The strains always cluster according to their PCR ribotype. The PCR-ribotype is indicated in brackets.
(EPS)

Figure S12 **RNA polymerase B gene-based dendrogram.** Neighbor joining dendrogram reflecting the similarity for all 48 analyzed *C. difficile* strains based on the gene for RNA polymerase B. The distance matrix was computed using the Hamming distance with the RNA polymerase B genes from all 48 *C. difficile* isolates. The strains always cluster according to their PCR ribotype. The PCR-ribotype is indicated in brackets.
(EPS)

Figure S13 **RNA polymerase C gene-based dendrogram.** Neighbor joining dendrogram reflecting the similarity for all 48 analyzed *C. difficile* strains based on the gene for RNA polymerase C. The distance matrix was computed using the Hamming distance with the RNA polymerase C genes from all 48 *C. difficile* isolates. The strains always cluster together according to their PCR ribotype. The PCR-ribotype is indicated in brackets.
(EPS)

Figure S14 **Dendrogram based on the concatenated sequences of the 14 conserved genes.** Neighbor joining dendrogram reflecting the similarity for all 48 analyzed *C. difficile* strains based on the concatenated sequences of the 14 conserved genes. The distance matrix was computed using the Hamming distance with these sequences from all 48 *C. difficile* isolates. The strains always cluster according to their PCR ribotype. The PCR-ribotype is indicated in brackets. Strains with ribotype 027 build two sub-clusters.
(EPS)

Figure S15 **Binary toxin A gene-based dendrogram.** Neighbor joining dendrogram reflecting the similarity for all 22 analyzed *C. difficile* strains based on the gene for binary toxin A. The distance matrix was computed using the Hamming distance with the binary toxin A genes from all 22 *C. difficile* isolates. The strains always cluster according to their PCR ribotype. The PCR-ribotype is indicated in brackets.
(EPS)

Figure S16 **Binary toxin B gene-based dendrogram.** Neighbor joining dendrogram reflecting the similarity for all 42 analyzed *C. difficile* strains based on the gene for binary toxin B. The distance matrix was computed using the Hamming distance with the binary toxin B genes from all 42 *C. difficile* isolates. The strains always cluster according to their PCR ribotype. The PCR-ribotype is indicated in brackets.
(EPS)

Figure S17 **Binary toxin R gene-based dendrogram.** Neighbor joining dendrogram reflecting the similarity for all 42 analyzed *C. difficile* strains based on the gene for binary toxin R. The distance matrix was computed using the Hamming distance with the binary toxin R genes from all 42 *C. difficile* isolates. The strains always cluster according to their PCR ribotype. The PCR-ribotype is indicated in brackets.
(EPS)

Figure S18 **Toxin C gene-based dendrogram.** Neighbor joining dendrogram reflecting the similarity for 47 *C. difficile* strains based on the gene for toxin C. The distance matrix was computed using the Hamming distance with the toxin C genes from the 47 *C. difficile* isolates containing this gene. The strains always cluster according to their PCR-ribotype. The PCR-ribotype is indicated in brackets.
(EPS)

Table S1 **Distance Matrices of all analysed genes.**
(XLSX)

Table S2 **Listing of all identified specific genes in every comparison.**
(XLSX)

Table S3 **Listing of the number of all computed core-genomes and specific genes.**
(XLSX)
